# Case of Catalepsy, with Convulsions of the Right Arm

**Published:** 1845-01

**Authors:** John W. Irby

**Affiliations:** Blacks and Whites, Nottoway County, Virginia


*Case of Catalepsy, with convulsions of the right arm.* By John W. Irby, M. D., of Blacks and Whites, Nottoway County, Virginia.

Communicated in a letter to the Editor.

A young woman, naturally healthy and of strong constitution, gave birth to her fifth child on the 27th of November last, after a natural labour. She did well until the 3rd of December, when she was attacked with symptoms "of phlegmasia dolens," a disease under which she had laboured in a former confinement. I saw her on the 5th: she then had no fever, nor had she had any of consequence. I directed 6 grs. of calomel with dover's powder, to be repeated in five hours, and flannel wrung out of hot vinegar and salt, to be applied every hour to the effected limb. On the morning of the 6th she took a dose of castor oil, which operated well, and she felt much better. A continuation of the fomentations, and oil when necessary, with bland diet, relieved all the symptoms of plegmasia dolens in a few days, so that on the 9th, 10th and 11th, she expressed herself as feeling entirely well except an occasional headache. I saw her on the morning of the 11th, and she was then up, walking about her room, and continued up all the day, occupied in sewing or knitting: her bowels were in good order, and she said the cold in her head was better, and that she was very well except a slight soreness in the back of her neck: during the day she had complained of a darting pain in her jaws, which lasted but a short time. That evening (11th) while sitting by the fire knitting, she got up to walk across the floor, and fell suddenly, saying that her left side was dead, (it was the right leg that was affected with phlegmasia dolens.) I saw her a few minutes after; she was on her back, breathing naturally, her pulse not disturbed, her head thrown back and her eyes turned upwards; the pupils dilated, the iris not affected by light; her whole left side as cold as marble; the leg and arm of that side perfectly stiff and defying any attempt to bend them; her *right* arm moving incessantly, like one beating eggs. She was speechless, and I suppose insensible; showing no signs of recognition, hearing or feeling. I gave her an emetic, which, as she appeared not to know that any any thing was in her mouth, I had much difficulty in getting down; it acted well, but during its operation she did not change the position of her head, nor did her eyes move.

Although her bowels had been moved twice during the day, I gave her a dose consisting of 15 grains each of jalap and rhubarb, with 10 grains of calomel; hoping that by a strong impression on her bowels, the great disturbance of nervous function might be relieved; and also applied cups to the temples and nape of the neck, and blisters to the calves of the legs.

Morning of the 12th. Symptoms the same; has not moved any part of her body except the right arm, which continues to go as if propelled by steam; pulse and respiration natural; medicine not having operated, ordered turpentine injections, which procured one stool; blisters drew well; applied cups again to temples and neck; by night, arm became still, but paralyed; never flinched while dressing the blisters; pulse natural and breathing good; pupils contract slightly on the approach of light; upper extremities and left leg stiff.

13th. Pupils more sensible to light; winks her eyes occasionally, pulse and breathing natural; injection of senna and salts, which operated well; took a little nourishment with much difficulty; rubbed mercurial ointment in the groins and axilla.

14th. Pupil very sensitive to light; pulse and breathing good; continued mercurial ointment. 9 P. M., feet and hands cold; dripping perspiration; pulse frequent and feeble; dressed her blisters with quinine, and administered stimulants, which kept her alive until the night of the 15th, when she expired.

*December* 23, 1844.

